# Supplementary figures and images for: A machine learning and network framework to discover new indications for small molecules
Source: PLoS Comput Biol. 2020 Aug 7;16(8):e1008098. doi: 10.1371/journal.pcbi.1008098 (PMC7437923; doi:10.1371/journal.pcbi.1008098)

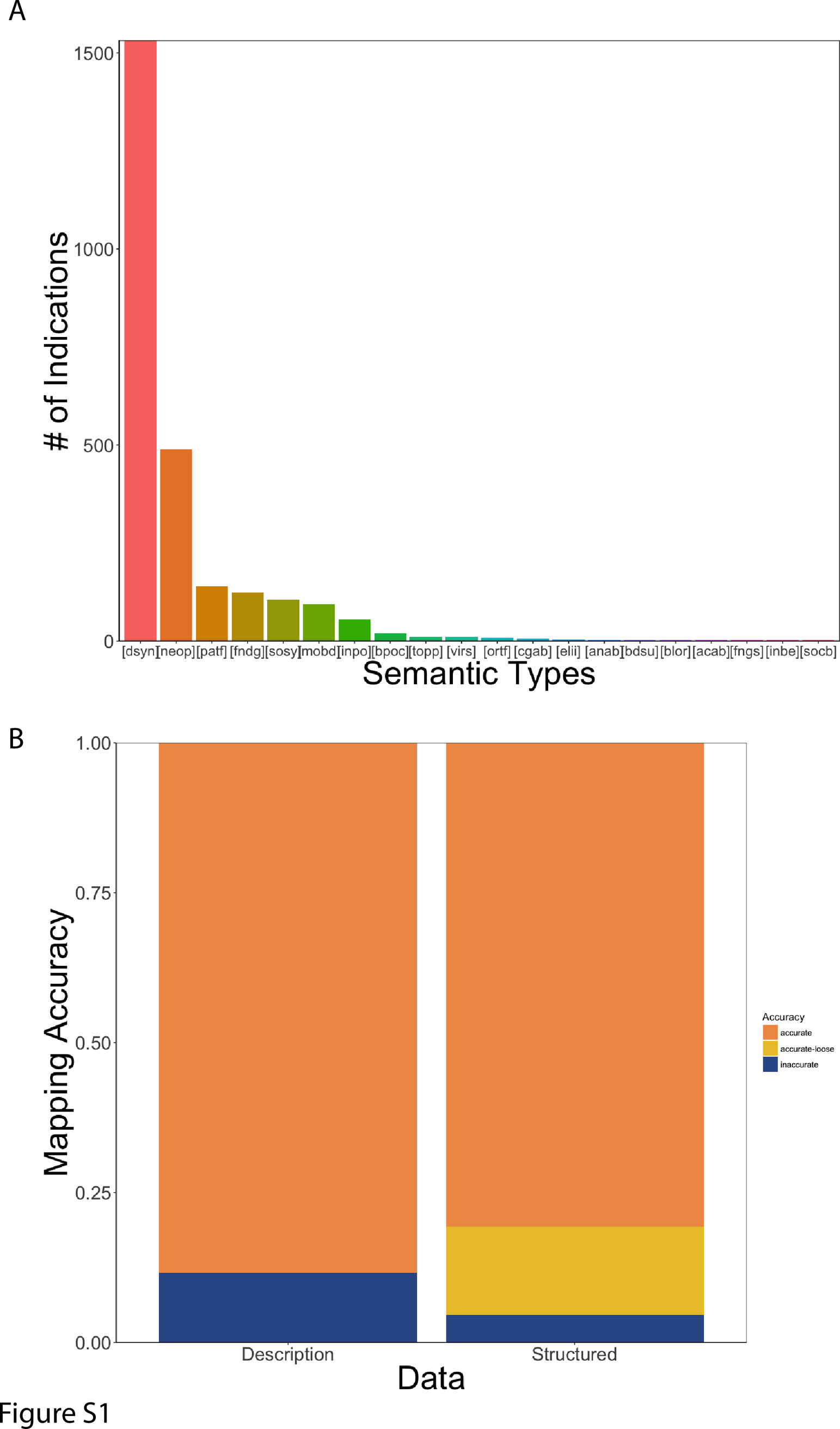

Supplement: S1 Fig — A) The number of occurrences of different UMLS sematic types. B) The accuracy of mapping indications using MetaMap for indications categorized a “Structured” and the “Description” section. (TIF) [file pcbi.1008098.s001.tif]

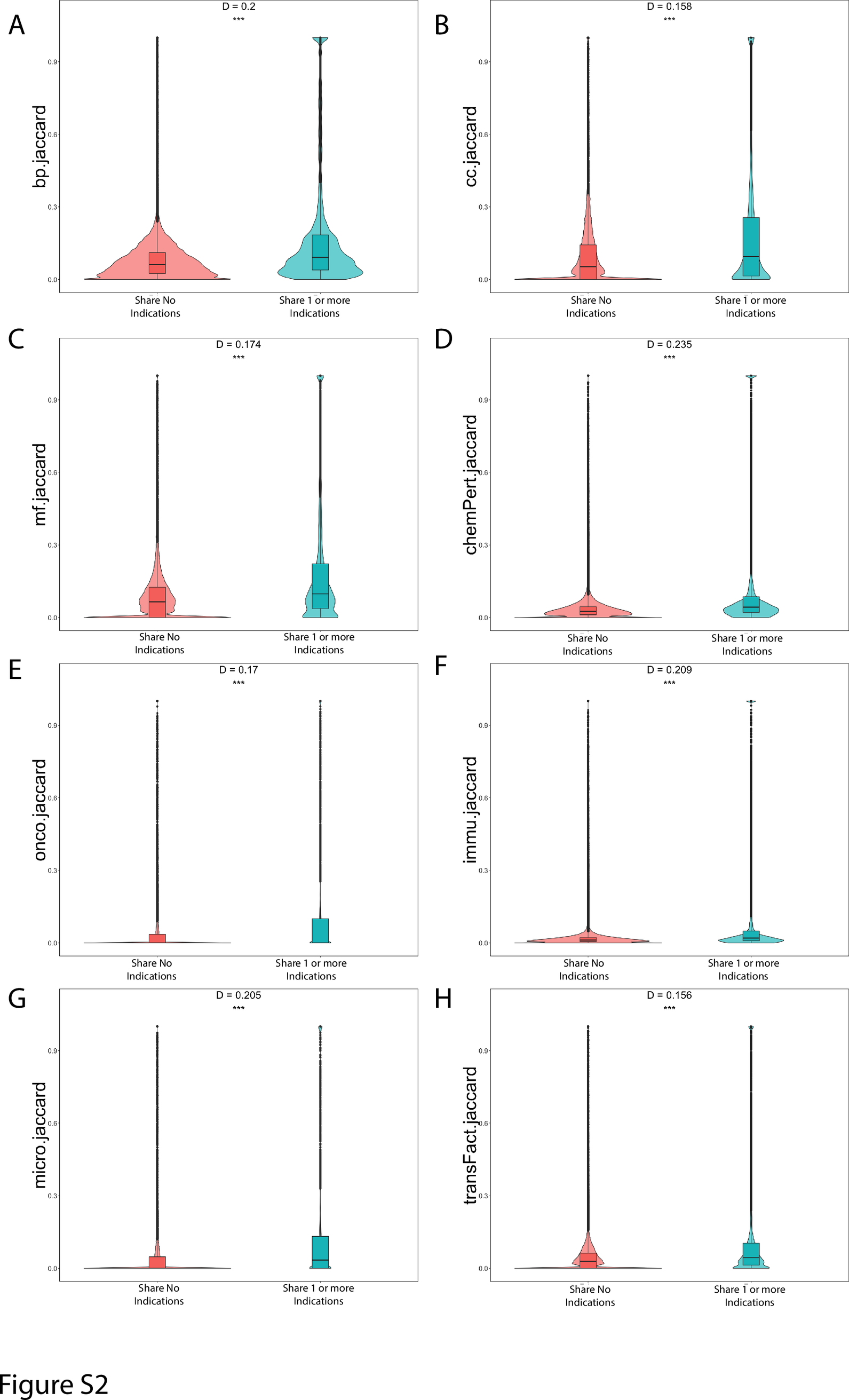

Supplement: S2 Fig — The violin plots of similarity distributions for the similarities of targets’ A) biological processes, B) cellular component, C) molecular function, D) chemical perturbation, E) oncological, F) immunogenic, G) micro-RNA, and H) transcription factor. Statistical significance found by Kolmogorov-Smirnov test. (TIF) [file pcbi.1008098.s002.tif]

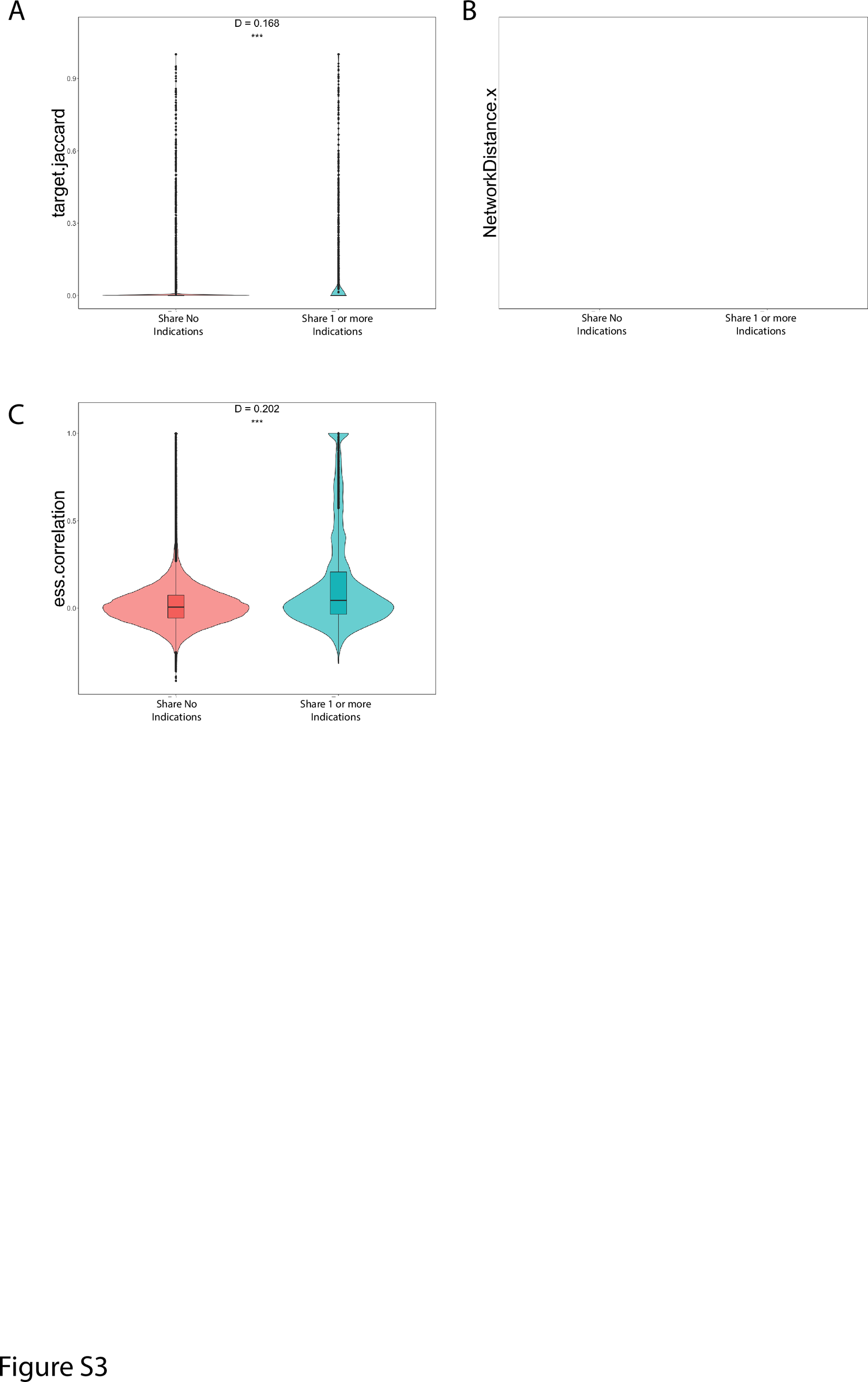

Supplement: S3 Fig — The violin plots of similarity distributions for the similarities of A) targets, B) the Protein-Protein Interaction network distance between targets and the C) correlation of target essential within cancer cell lines. Statistical significance found by Kolmogorov-Smirnov test. (TIF) [file pcbi.1008098.s003.tif]

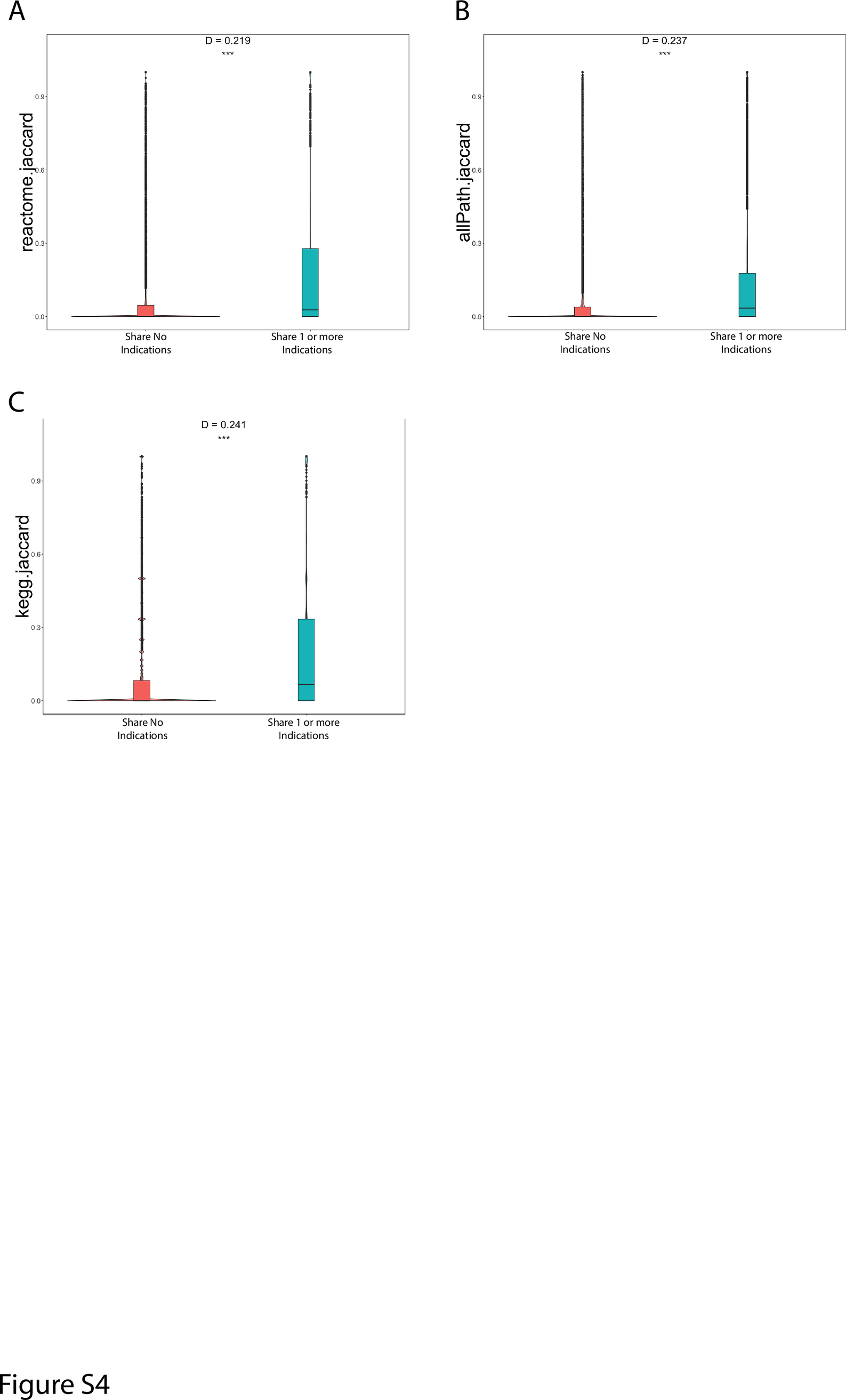

Supplement: S4 Fig — The violin plots of similarity distributions for the similarities of the A) reactome pathways, B) all pathway types and C) KEGG pathways a drug’s target is known to be involved within. Statistical significance found by Kolmogorov-Smirnov test. (TIF) [file pcbi.1008098.s004.tif]

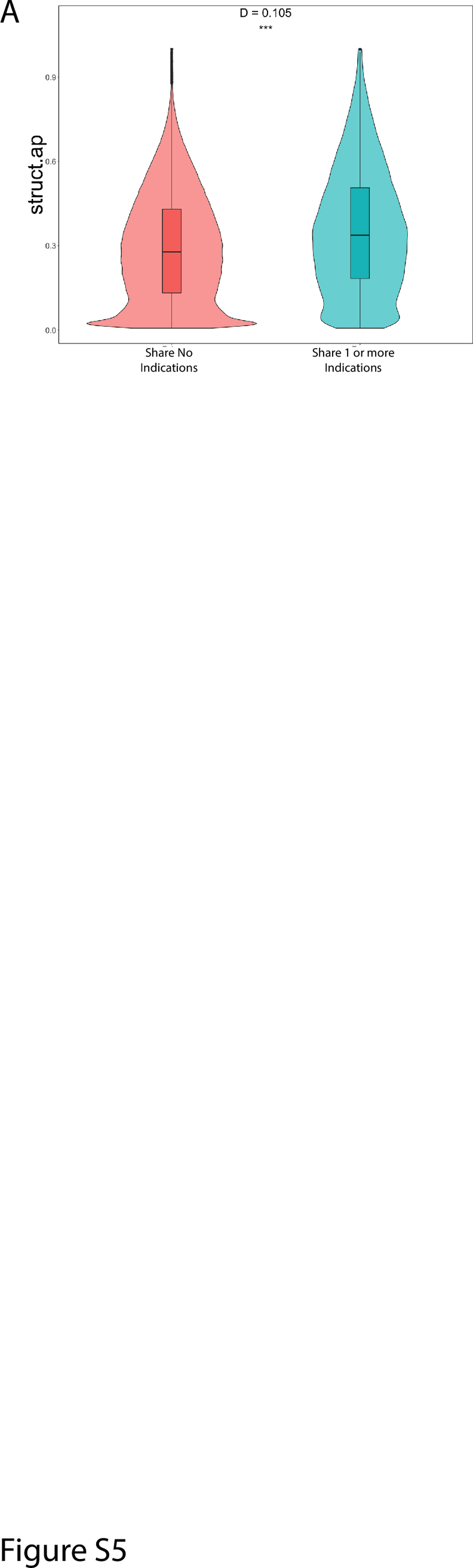

Supplement: S5 Fig — A) The violin plot of the Dice chemical fingerprint similarity, statistical significance found by Kolmogorov-Smirnov test. (TIF) [file pcbi.1008098.s005.tif]

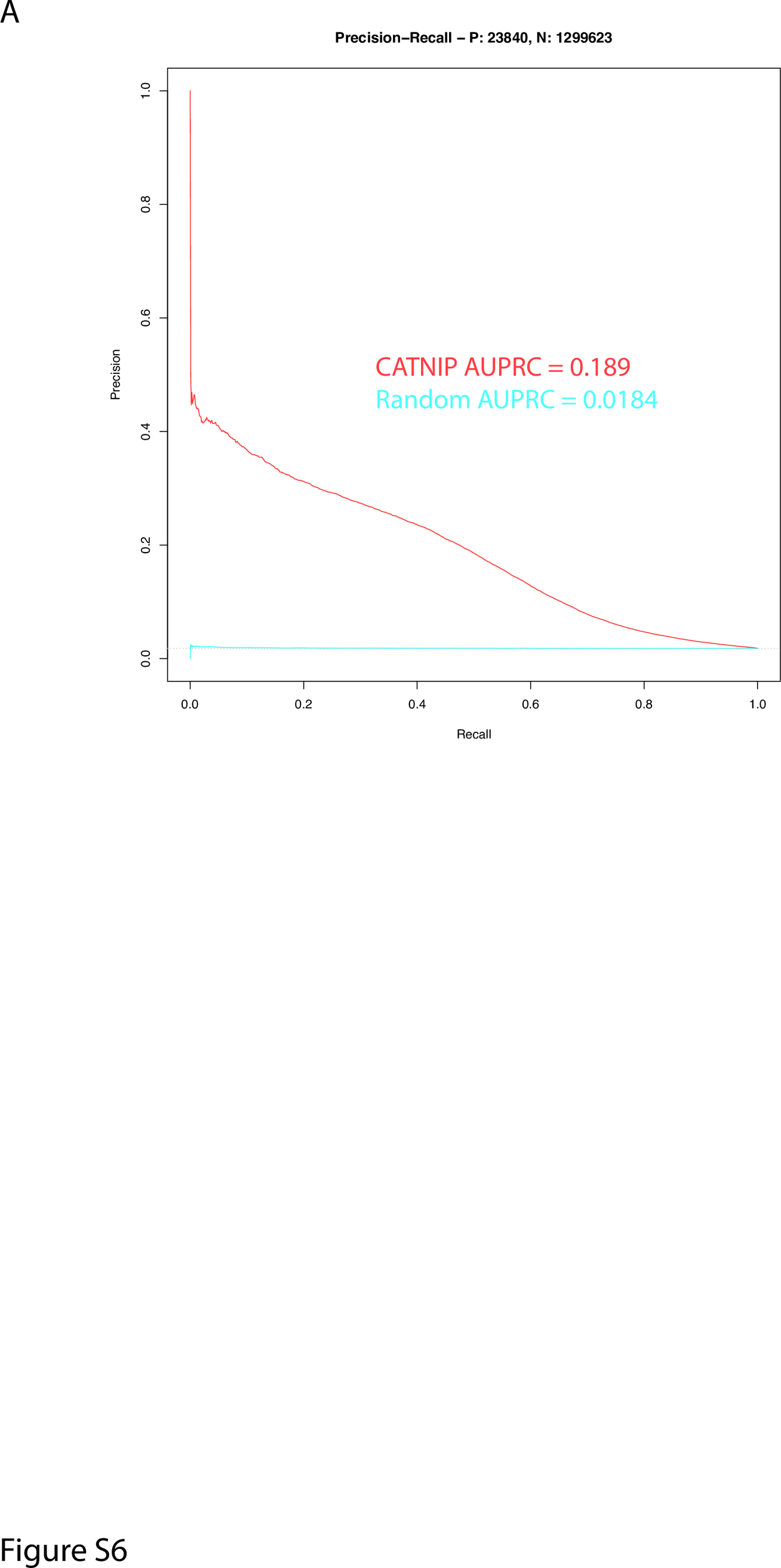

Supplement: S6 Fig — A) The Precision–Recall curve for classifying if two drugs share an indication using CATNIP and the random expectation. (TIF) [file pcbi.1008098.s006.tif]

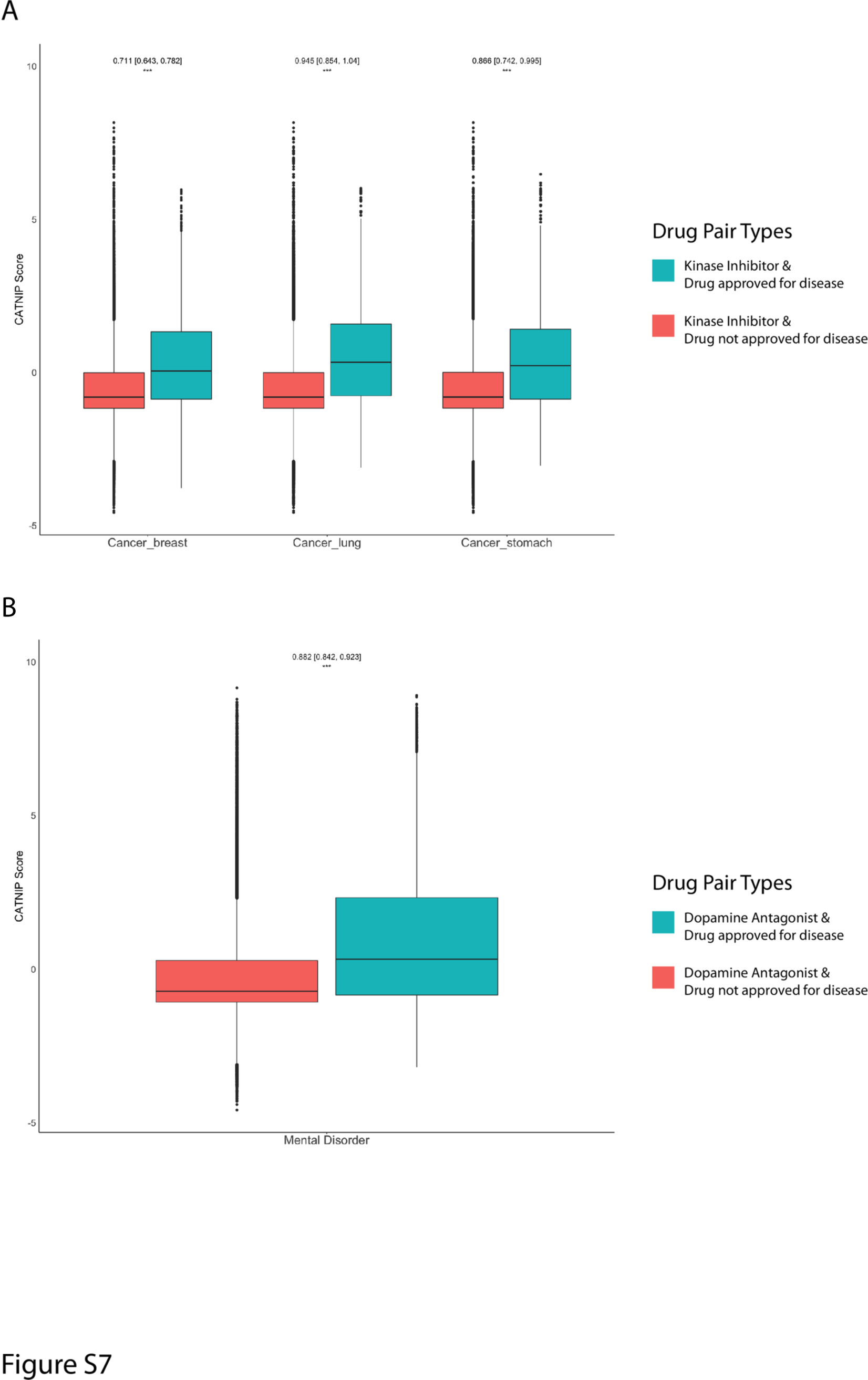

Supplement: S7 Fig — The distributions of CATNIP score between A) kinase inhibitors and drugs known to treat cancer and those that do not and B) dopamine antagonists and drugs known to treat mental illness and those that do not. (TIF) [file pcbi.1008098.s007.tif]

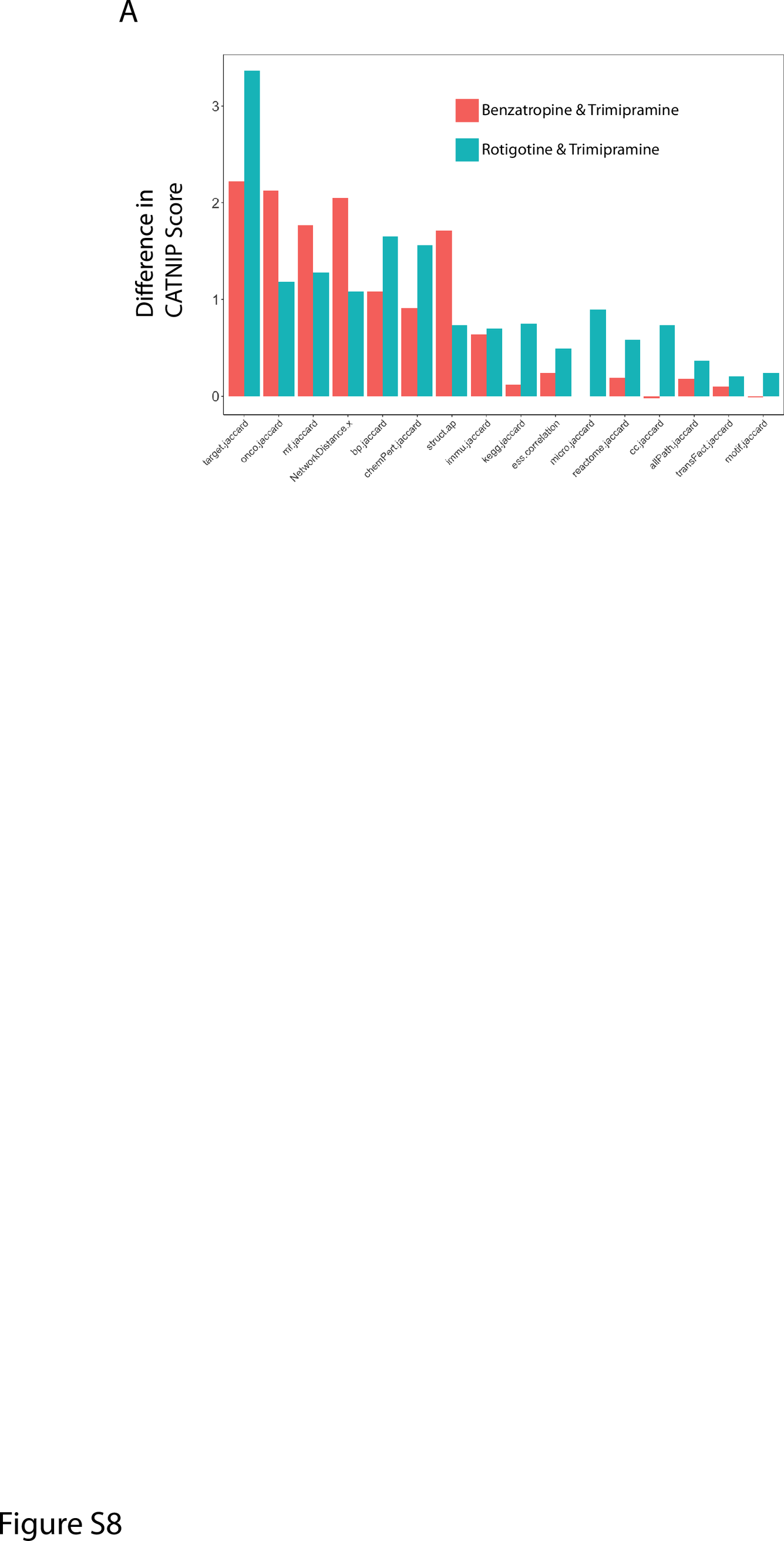

Supplement: S8 Fig — A) The decrease in the CATNIP score when removing each feature for trimipramine and select Parkinson’s Disease drugs. (TIF) [file pcbi.1008098.s008.tif]

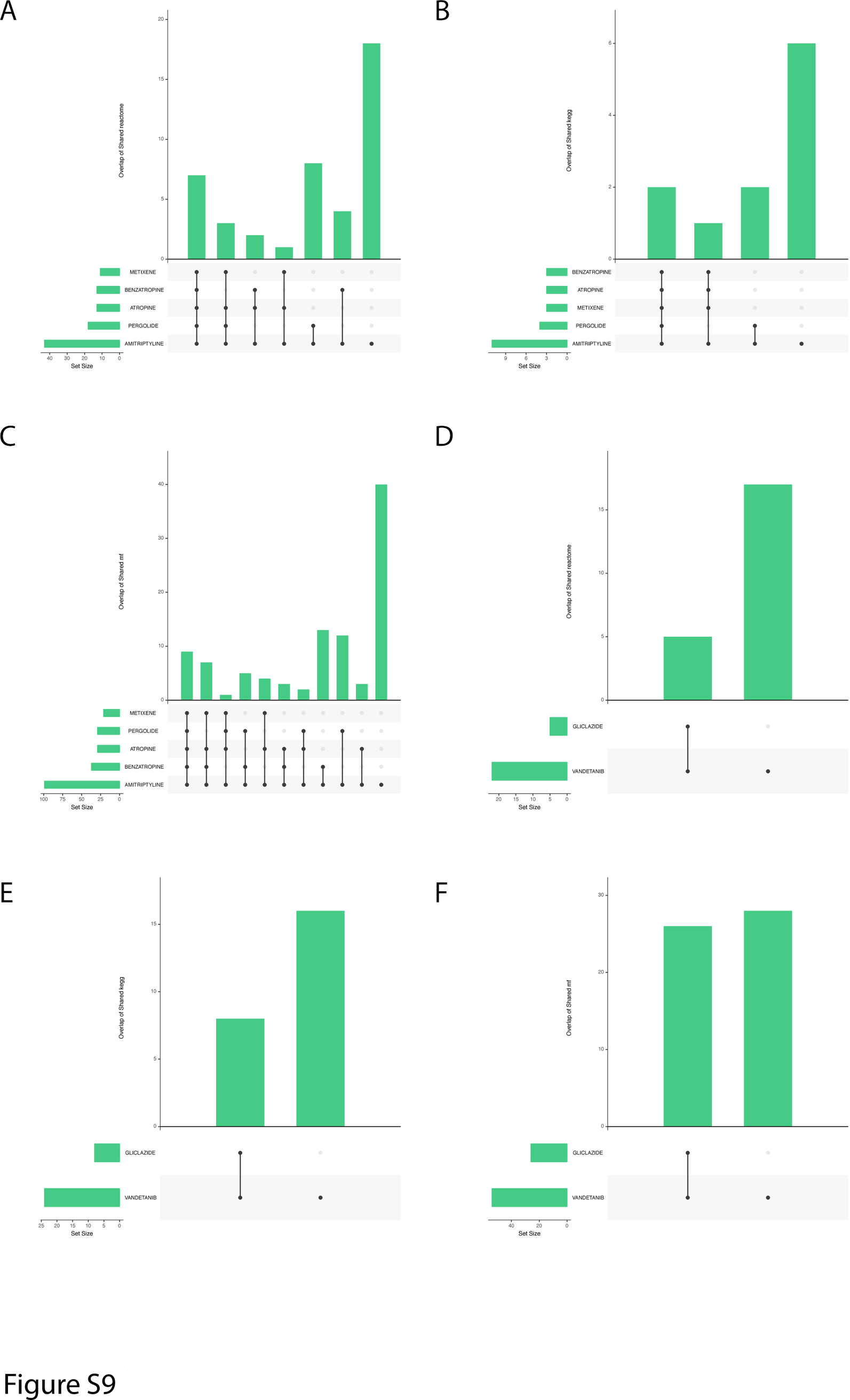

Supplement: S9 Fig — The overlap between amitriptyline and select Parkinson’s Disease drugs for A) reactome pathways, B) KEGG pathways, and C) molecular function gene ontologies. The overlap between vandetanib and gliclazide for D) reactome pathways, E) KEGG pathways, and F) molecular function gene ontologies. (TIF) [file pcbi.1008098.s009.tif]

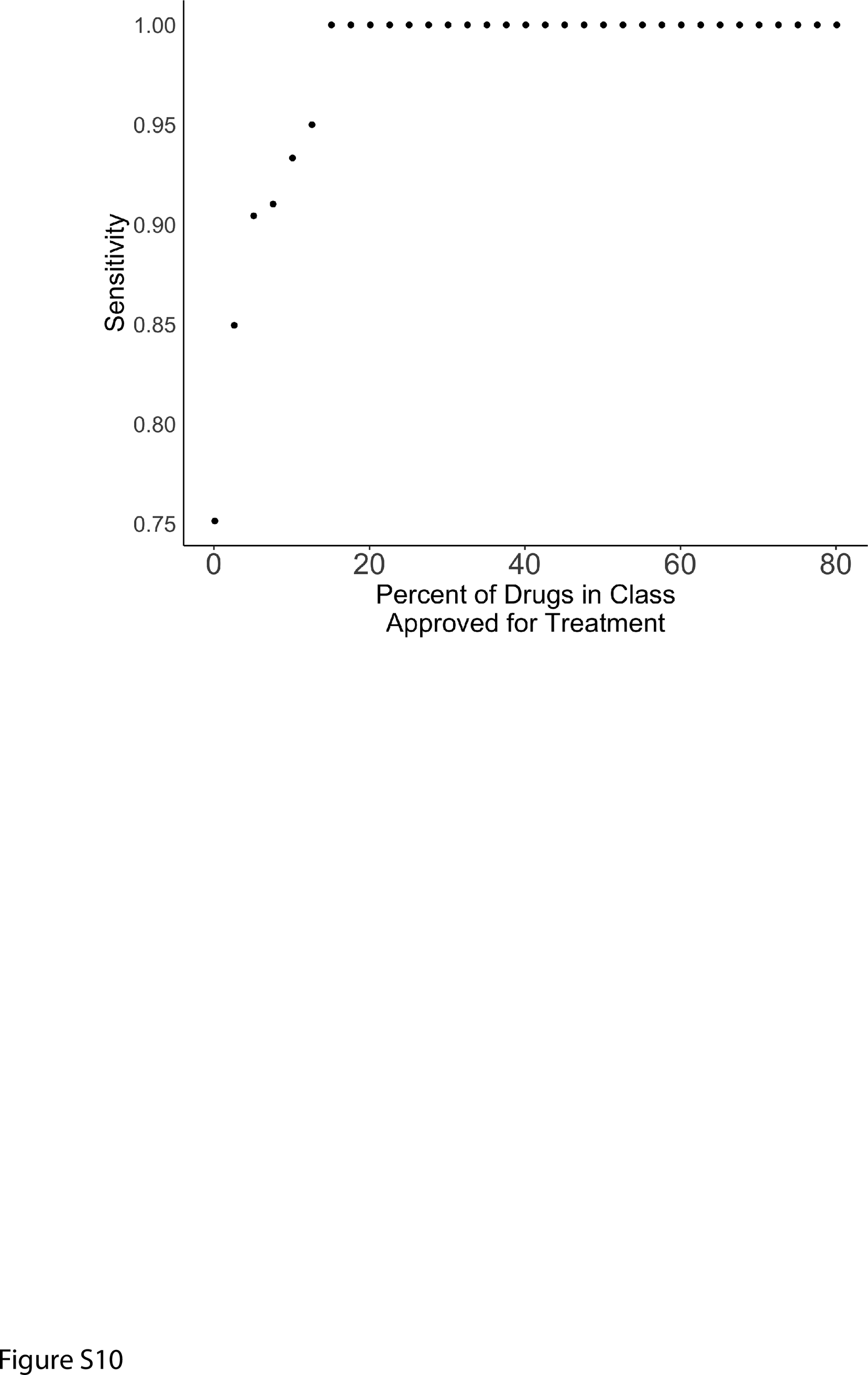

Supplement: S10 Fig — (TIF) [file pcbi.1008098.s010.tif]

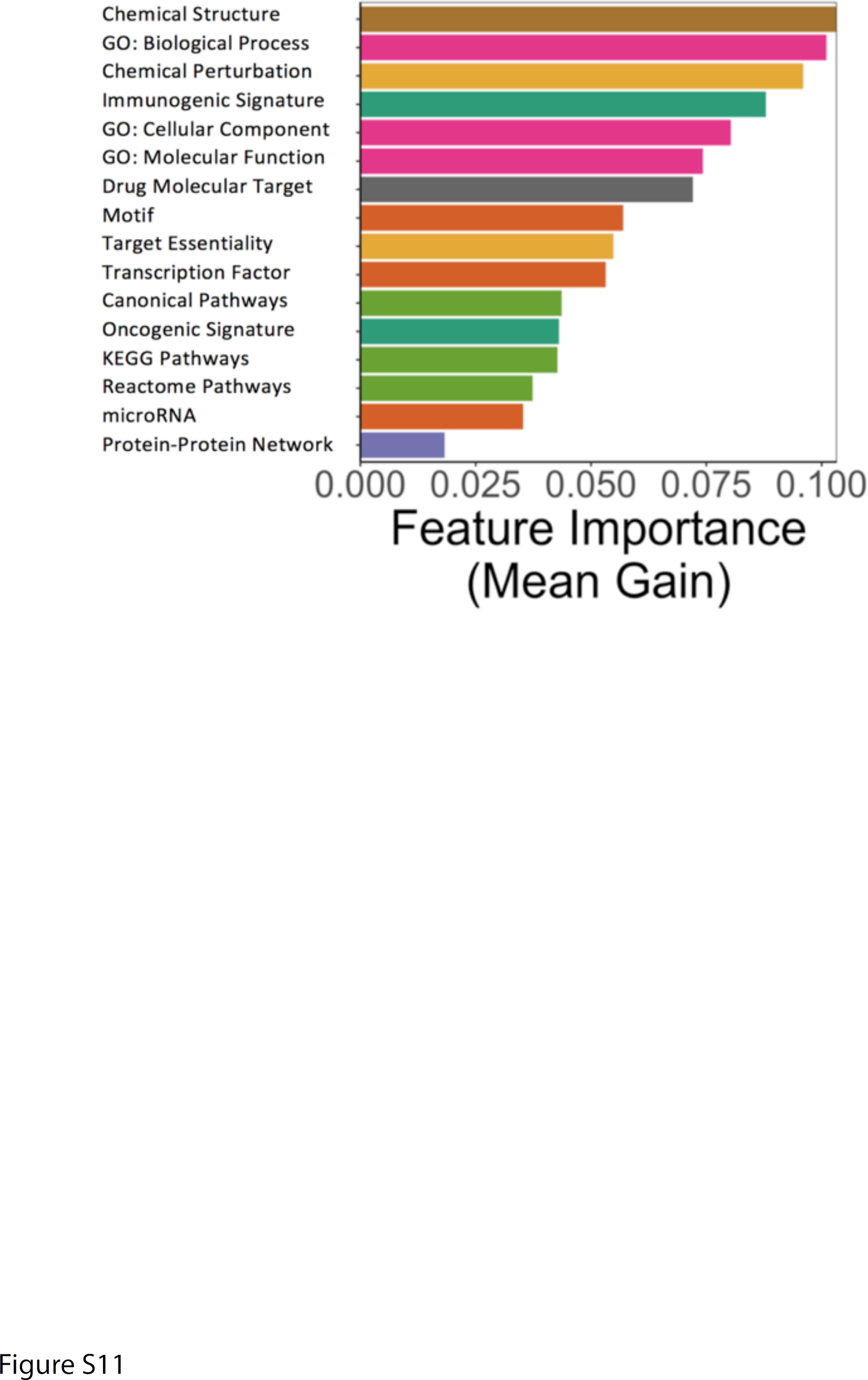

Supplement: S11 Fig — (TIF) [file pcbi.1008098.s011.tif]

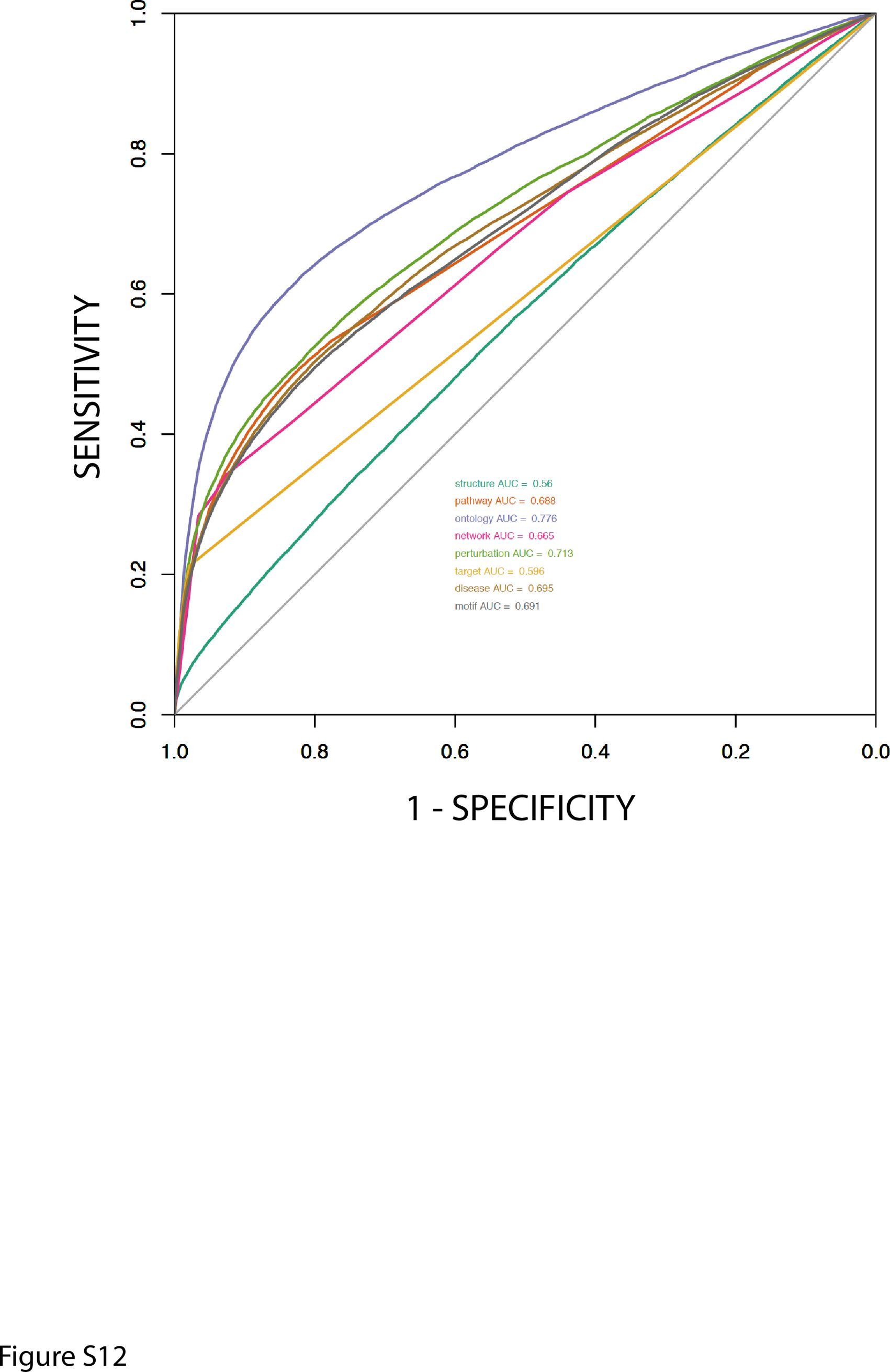

Supplement: S12 Fig — (TIF) [file pcbi.1008098.s012.tif]
