# Supplementary material for: A machine learning and network framework to discover new indications for small molecules
Source: PLoS Comput Biol. 2020 Aug 7;16(8):e1008098. doi: 10.1371/journal.pcbi.1008098 (PMC7437923; doi:10.1371/journal.pcbi.1008098)
Supplement: S1 Methods — (DOCX) [file pcbi.1008098.s017.docx]

**Supplementary Methods**

***Comparison with PREDICT***

The method PREDICT by Gottlieb (2011) was implemented, which measures the similarity of a drug-disease to the nearest known drug-disease pair, with a heavily curated dataset. This method generates a score based on the geometric mean combining drug-drug similarities and disease-disease similarities. When implementing PREDICT, we tested it against our method by focusing on drug similarity information, removing drug-disease associations from their model. This allowed us to properly compare our classification results, as we applied PREDICT in a modified setting in which disease information was not available. Additionally, to determine the AUC, we restricted to only diseases that had UMLS Concept Unique Identifiers (CUIs).
